# Retrospective Wastewater Tracking of Measles Outbreak in Western Switzerland in Winter 2024

**DOI:** 10.1021/acs.estlett.5c00244

**Published:** 2025-05-22

**Authors:** Charles Gan, Melissa Pitton, Jolinda de Korne-Elenbaas, Ludovico Cobuccio, Alessandro Cassini, Christoph Ort, Timothy R. Julian

**Affiliations:** † 28499 Eawag, Swiss Federal Institute of Aquatic Science and Technology, Dübendorf 8600, Switzerland; ‡ Cantonal Doctor Office, Public Health Department, Lausanne 1014, Switzerland; § Infectious Diseases Service, Lausanne University Hospital, Lausanne 1005, Switzerland; ∥ Cantonal Office of Health, Geneva 1207, Switzerland; ⊥ Swiss Tropical and Public Health Institute, Allschwil 4123, Switzerland; # University of Basel, Basel 4001, Switzerland

**Keywords:** Measles, Wastewater surveillance, Digital PCR, Outbreak detection, Strain differentiation

## Abstract

Measles outbreaks remain a significant public health challenge, despite high vaccination coverage in many regions. Wastewater-based surveillance (WBS) offers a noninvasive and community-level approach to monitoring the circulation of pathogens, including the measles virus. Here, we retrospectively applied a duplex digital PCR assay to distinguish between wild-type and vaccine strains of the measles virus in wastewater samples available from an existing national WBS program. Samples originated from the wastewater treatment plant serving the Lausanne city catchment area, where an outbreak occurred before spreading to the broader Canton Vaud region. Despite high vaccination rates, viral loads of the measles wild type were detected during the first transmission event involving 21 cases identified within a week. However, viral loads were no longer detectable after the initial 21 cases, despite an additional 30 cases reported in the following 3 weeks, possibly due to lower incidence rate or location outside the catchment. Measles vaccine strain was not detected during the outbreak. Our results demonstrate the complementarity of WBS with clinical surveillance and suggest its potential as an early warning system for measles and other vaccine-preventable diseases. Further improvements in the assay sensitivity and integration with epidemiological data could enhance the utility of WBS for outbreak detection and control.

## Graphical abstract

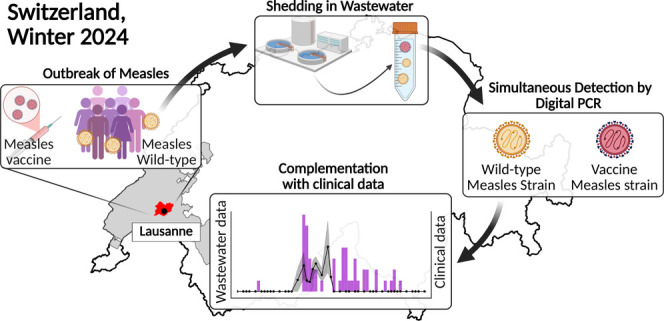

## Introduction

Measles is a highly contagious disease caused by the measles virus that can lead to complications such as pneumonia, encephalitis, and, in some cases, death.^1^ Measles virus is characterized by a basic reproduction number (R0) estimated to be around 15.7, significantly higher than that of many other infectious diseases.^2^ This high R0 underscores the rapidity with which the virus can spread in susceptible populations, making effective control measures crucial. A safe and highly effective vaccine has existed since the 1960s.^3^ However, measles remains a public health concern in many parts of the world due to waning vaccination coverage.^4^ Further, there have been multiple instances of vaccine breakthrough^5^ where people are infected despite previous vaccination. This breakthrough allows outbreaks to occur despite high vaccine coverage (>95% for herd immunity^6^). A recent example was observed in the Canton of Vaud (Switzerland),^7^ where the two-dose vaccination rate was high (91% for 2-year-olds, 94% for 8-year-olds, and 96% for 16-year-olds in 2023 ^8^), but still 51 confirmed cases emerged, of which 73% were vaccinated.^7^

Wastewater-based surveillance (WBS) has surfaced as a transformative tool for public health, providing an innovative approach to monitor pathogens at the community level. By analyzing wastewater for genetic material from viruses, WBS offers a cost-effective and noninvasive means to detect and track disease spread in alignment with reported clinical cases. This methodology has gained significant attention during the COVID-19 pandemic, where it proved to be instrumental in monitoring SARS-CoV-2 trends in real time. Beyond acute and novel outbreaks, WBS has also demonstrated utility in tracking endemic pathogens and providing early warnings for re-emerging diseases (e.g., polio).^9,10^ Furthermore, WBS could help detect breakthrough cases of diseases like measles,^11^ which, although rare, can occur even in highly vaccinated communities due to waning immunity or other factors.

Measles virus has been shown to be detectable in wastewater across multiple studies,^12–14^ particularly in catchments with reported clinical cases^12,13^ or symptomatic individuals.^14^ WBS of measles has also demonstrated its utility in populations with predominantly low vaccination coverage^12^ as well as those with more variable vaccination statuses.^13,14^ These findings highlight the potential of WBS to monitor the circulation of measles virus and track disease dynamics within communities. Furthermore, detection of strain-specific mutations with digital PCR (dPCR) enables differentiation between measles virus shed from community infections and that shed after vaccination, further enhancing the precision of wastewater surveillance.

From January through March 2024, a measles outbreak initiated in Lausanne city (within Canton Vaud, Switzerland) led to 51 reported clinical cases, of which at least 31 were breakthrough cases among people previously vaccinated with two doses.^7^ Coincident analyses of wastewater for circulating endemic respiratory pathogens^15^ allowed retrospective analysis of archived wastewater extracts for detection and quantification of measles. A duplex dPCR assay, with primer and probe sequences originally developed by Wu et al.,^16^ targeting and differentiating between wild-type (WT) measles and measles vaccine (VA) was applied using a different dPCR platform to evaluate WBS for tracking dynamics of measles virus circulation in the community. This approach provides a model for integrating wastewater data into measles control strategies.

## Methods and Materials

### Wastewater Composite Samples

Corresponding to the most recent measles outbreak which originated in Lausanne city and spread within the Canton of Vaud region between January and March 2024, 64 24 h composite influent samples from the wastewater treatment plant (STEP Vidy) covering the entire Lausanne city catchment area (population: 240,000) were measured for measles WT and VA between January 2nd and March 31st, 2024 (Table S1). All samples were collected in the context of an ongoing campaign to monitor respiratory viruses in Swiss wastewater.^15^ Samples were stored at 4 °C and then concentrated and extracted within 1 week of collection (2–7 days) using the Wizard Enviro Total Nucleic Acid Extraction Kit (Promega, Wisconsin, USA) protocol with OneStep PCR Inhibitor Removal Kit (Zymo Research, California, USA) as described previously.^17^ Murine hepatitis virus (MHV) was used as a process control to assess viral recovery, as previously described.^15^ MHV was spiked into 61% of samples, and median recovery efficiency was determined to be 12.5%, with an interquartile range of 10.6–15.4%. Extracts were stored at −80 °C and analyzed between October 8th, 2024, and December 5th, 2024. Measured extracts were 3-fold diluted prior to dPCR analysis to minimize inhibition.

### Digital PCR Assay

A duplex dPCR assay was developed for the measles virus, enabling simultaneous differentiation and quantification of WT and VA strains. Primers and probe sequences were sourced from Wu et al.^16^ (Table S2) and were manufactured at Integrated DNA Technologies (IDT; Iowa, USA). Fluorophore for the measles WT probe was adapted to contain a Freedom Cy5.5 dye, and both measles WT and VA probes were adapted to contain an Iowa Black RQ Dark Quencher (Table S2).

PCR prereaction volume was 27 μL, containing 5.4 μL of sample template and 21.6 μL of Mastermix. Mastermix consisted of qScript XLT 1-step RT-PCR Tough Mix (2× concentrated, Quantabio, Massachusetts, USA), 500 nM forward primer, 500 nM reverse primer, 200 nM WT probe, 200 nM VA probe, and RNase free water to fill. Primers and probe concentrations were adjusted from Wu et al.^16^ (900 nM to 500 nM for primers and 250 nM to 200 nM for probes) in line with manufacturer guidelines to minimize primer/probe interactions (Text S1, Figure S1). Of the total 27 μL of PCR prereaction, 25 μL was pipetted into the chamber of the Sapphire chip (Stilla Technologies, Villejuif, France). The chip was then placed inside of a Geode (Stilla Technologies, Villejuif, France) for partitioning and thermocycling with the following steps: partitioning for 12 min at 40 °C, reverse transcription at 50 °C for 1 h, enzyme activation at 95 °C for 10 min, then 40 cycles of denaturation at 94 °C for 30 s and annealing/extension at 55 °C for 1 min, then finally an end cycle pressure release was added according to the manufacturer’s instructions.

The measles WT/VA duplex, originally implemented on a Biorad QX600 droplet dPCR system by Wu et al.,^16^ was translated onto the Naica system 6-color digital PCR system (Stilla Technologies, Villejuif, France) by modifying probe dyes and quenchers, primer and probe concentrations, polymerase, reverse transcriptase, and sample input volume according to manufacturer recommendations. To validate performance in our lab, the duplex assay was optimized using two types of positive controls: (i) live-vaccine strain for measles (Cat No. M0210000), provided by European Directorate for the Quality of Medicines & HealthCare extracted with the QiaAmp Viral RNA MiniKit (Qiagen, Hilden, Germany) following manufacturer instructions, and (ii) a synthetic DNA construct for the most recent circulating genotype of measles (i.e., B3 genotype) (gBlock, IDT, Iowa, USA) (Table S3). The assay was first validated as a single-plex assay (Text S2) and then validated as a duplex assay (Text S3) to ensure no deviations were present when multiplexing (Figure S2). Validation consisted of testing the linearity of three 10-fold dilutions in triplicate (Figure S3).

All wastewater samples were run in duplicate and with a positive control containing both the measles WT and measles VA strains (Table S3). A no-template control (NTC) was also run simultaneously, and all experiments with more than two positive partitions within the NTC were rerun. Partitions were classified on Crystal Miner Software version 4.0 (Stilla Technologies) by using polygon gating (Figure S3). Chambers with less than 15,000 droplets were not considered and corresponding samples were assayed again. Digital minimum information for publication of quantitative real-time PCR experiments (dMIQE) guidelines for the extracts used are mentioned elsewhere.^15^

PCR inhibition by wastewater matrix was quantified according to a method described previously.^15,18^ Synthetic DNA of Measles B3 (gBlock, IDT, Iowa, USA) was spiked in a random subset of the wastewater extracts used to generate the time series. Inhibition, expressed in percentage, was defined as one minus the ratio between the amount measured in a spiked extract divided by the amount spiked plus the endogenous measles WT within the extract. Inhibition ranged from 0 to 100%, with 0 indicating no inhibition and 100% implying a completely inhibited sample. Samples with inhibition greater than 40% were considered inhibited and rerun at a higher dilution.

### Assay Sensitivity

We first defined a sample as positive or above the Limit of Blank (LoB) (i.e., target present with 95% confidence that it is not random noise) if there were three or more positive partitions (i.e., droplets), which corresponds to approximately 5 genome copies per reaction, as described previously by Nyaruaba et al.^19^ Limit of detection (LoD) and quantification (LoQ) were identified by measuring decreasing concentrations with dPCR and by model fitting.^20^ LoD, representing an assessment of the sensitivity of our assay, was defined as the lowest concentration above LoB at which a sample would be detected with 95% confidence. The LoQ, which is the concentration at which the quantitative estimate of a concentration is a reliable estimate, was defined here as the concentration at which the coefficient of variation (CoV) was 30%, in line with recommendations from the literature.^21,22^ Additional details regarding the determination of LoD and LoQ are provided in Text S4.

### Clinical Data

Data about clinical confirmed cases, including the number, timing, and location of measles cases from people with different immunization status (fully immunized referring to 2 doses of measles-containing vaccine (MCV) or previous measles infection, partially immunized referring to 1 dose of MCV, or not immunized referring to no dose of MCV and no previous infection) was sourced from Cassini, Cobuccio, et al.^7^

## Results and Discussion

### Detection of Measles RNA in Wastewater Samples from Lausanne City

We applied a duplex digital PCR assay capable of differentiating measles WT and VA strains to retrospectively analyze a total of 64 wastewater samples. Of these, we detected viral loads of the measles WT in nine samples (14%) that exceeded our defined LoB (Figure 1). Of these nine positive samples, two (22%) surpassed the two-replicate LoD of 20,600 gc/L_ww_ (Figure 1). None of the positive samples exceeded the determined LoQ of 153,600 gc/L_ww_. For the quantification of measles VA, all samples were below the established LoB, determined two-replicate LoD (11,100 gc/L_ww_), and determined LoQ (28,900 gc/L_ww_) and are omitted from further analysis. Modeled LoD and LoQ curves are provided in the Supporting Information (Figures S4 and S5).

**Figure 1:**
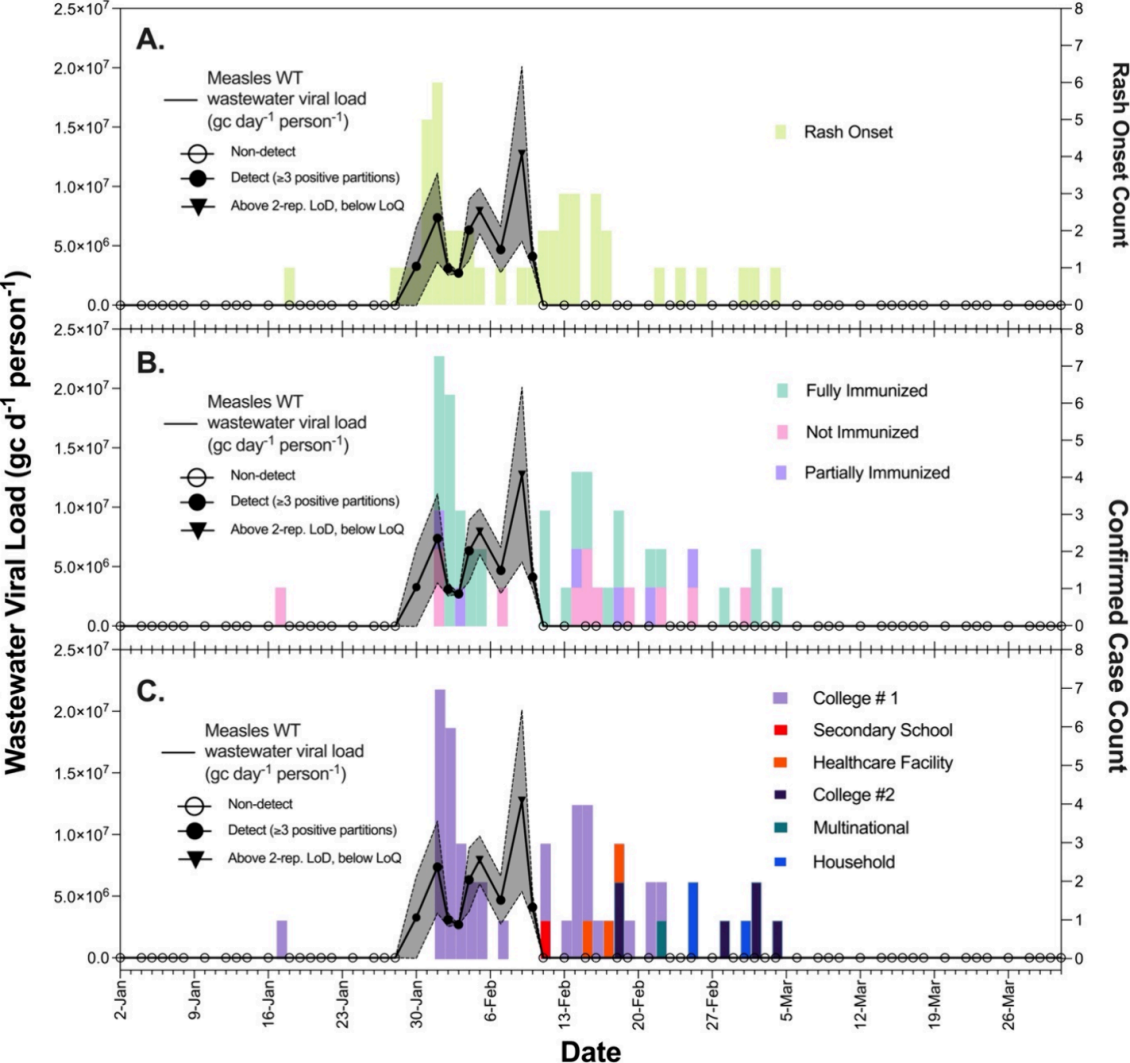
Temporal trends in measles viral load and rash onset and confirmed case counts from early January to late March 2024. (A–C) The primary vertical axis (left) shows the viral load in wastewater expressed as gene copies per day per person (gc d^–1^ person^–1^), represented by a black line with black open circles (below LoB, i.e. nondetected), black filled circles (above LoB, i.e. 95% confidence it is not random noise; at least three positive partitions), and black inverted triangles (above 2-replicate LoD but below LoQ, i.e. detected with 95% confidence when performing two replicates, >30% expected coefficient of variation) data points. The gray shaded region indicates the standard deviation around the mean of two replicate measurements of the viral load. The horizontal axis represents the date in 2024. (A) The secondary vertical axis (right) shows the number of people with rash onset observed and recorded by the hospitals in the area. (B) The secondary vertical axis (right) shows the number of confirmed measles cases by immunization type, depicted as stacked pink (not immunized), purple (partially immunized), and green (fully immunized) bars representing immunization status. (C) The secondary vertical axis (right) shows the number of confirmed measles cases by location, depicted as stacked purple (college #1), red (secondary school), orange (healthcare facility), dark purple (college #2), green (multinational), and blue (household). Clinical cases, their timing, and transmission chain analysis can also be viewed at https://www.cobuccio.me/measles_epicurve.html (last accessed: 14.03.2025) and https://www.cobuccio.me/transmission_chains_colorblind_proof (last accessed: 14.03.2025).

To assess potential PCR inhibition, we tested a subset of 10 samples (Table S1). We observed a maximum inhibition value of 9%, which remained well below our acceptable threshold of 40%, suggesting minimal inhibition. Peaks in the measles WT viral load were observed in early and mid-February. Afterward, we observed a decline in viral load below our detection threshold, which remained so throughout March. Absence of measles WT detection could be attributable to absence of shedding in wastewater or limited assay sensitivity, which could be improved by increasing the template input or the volume of processed wastewater, as done by Wu et al.^16^

Overall, the viral concentrations of measles WT (averaged across duplicates) ranged from 8.25 × 10^3^ gc/L_ww_ to 3.3 × 10^4^ gc/L_ww_ (Table S1), while the corresponding measles WT viral load (averaged across duplicates) ranged from 3.09 × 10^6^ to 1.27 × 10^7^ gc day^–1^ person^–1^ (Table S1). Despite a call for vaccination issued on February 2nd, 2024,^7^ we did not detect measles VA in any of the samples analyzed.

### Rash and Cases

Rash onset is a symptom-based metric which generally precedes laboratory-confirmed cases.^7^ Rash onset was recorded over a 46-day with 54% (*n* = 25) of the days having rash onset occurrences and 22% (*n* = 10) of days showing wastewater viral loads above the detection threshold (Figure 1A). Measles WT virus in wastewater aligned most closely with the reported rash onset occurring between January 28th and February 10th consisting of 25 counts (incidence rate: 1.79 rash count per day). No wastewater viral loads were detected at any other point, despite the presence of an additional 30 rash counts (incidence rate of 1.30 rash count per day) in the time period directly after from February 11th to March 4th.

For confirmed case counts, a similar pattern was observed where cases were recorded over 47 days with 47% (*n* = 22) having confirmed case counts and 21% (*n* = 10) having wastewater loads above the detection threshold. Measles WT virus in wastewater aligned most closely with the 21 confirmed cases occurring between February 1st and February 7th (incidence rate: 3 cases per day). Similar to rash onset counts, no viral load was detected at any other point despite the presence of an additional 30 cases (incidence rate: 1.15 cases per day) in the time period after from February 8th to March 4th.

These observations indicate that wastewater viral loads closely reflected the rash onset and reported cases during the early phase of the outbreak. The subsequent absence of a detectable measles WT signal in wastewater may be attributed to a decline in incidence, highlighting that a minimum number of infected individuals is required to exceed the detection threshold. We also highlight the differences in timing for symptom rash onset and case confirmation, underscoring the additional utility of a third source of information from WBS to complement. By capturing viral signals independent of clinical testing and potential reporting delays, WBS offers the potential to serve as an additional indicator for early warning.

### Implications of Vaccination on Wastewater Viral Loads

Within the initial cluster of 21 cases, 14% (*n* = 3) were not immunized (no prior infection, no MCV dose), 10% (*n* = 2) were partially immunized (one dose of MCV), and 76% (*n* = 16) were fully immunized (two doses of MCV or prior infection). In contrast, the later clusters comprising 30 cases had a higher proportion of not immunized individuals (27%, *n* = 8), with 14% (*n* = 4) partially immunized and 59% (*n* = 17) fully immunized (Figure 1B).

Although vaccination typically reduces disease severity and viral load,^5,23^ viral loads in wastewater were highest during the initial cluster, which had a higher proportion of immunized individuals. This observation could be due to the co-occurrence of breakthrough cases and higher incidence rate reflective of a higher initial attack rate, which could have led to increased shedding. Differently, later transmission clusters, which were not associated with detectable wastewater viral loads, had a higher proportion of not immunized individuals. We attribute this to the smaller scale of transmission and lower case and rash onset incidence rate.

Overall, these findings suggest that cases among immunized individuals can still contribute to community-level shedding, emphasizing the need for further research to quantify shedding dynamics across vaccination statuses. Specifically, shedding within urine is of particular interest, as it was recently identified by Chen and Bibby as the main contributor to shedding in wastewater.^11^ A recent study by Kurata et al. provided evidence that viral gene copies in urine specimens were a factor of 19.1 higher in individuals who are immunologically naïve versus those who had been previously vaccinated.^24^ However, as only two people were sampled in the naïve group, no significance could be determined. Other studies characterize gene copy concentration of measles virus in urine; however, they are limited to virus shed from the vaccine administration itself.^25–27^ Understanding how factors such as the number of vaccine doses influence shedding in breakthrough cases could provide valuable insights into the detectability of the measles virus in wastewater-based surveillance and its potential role in monitoring transmission dynamics.

### Influence of Spatial Factors on Wastewater Detection of Measles

The detected measles WT viral loads in wastewater coincided with cases primarily linked to “College #1”, a confirmed location within the catchment area monitored (Figure 1C). As viral loads became undetectable, cases associated with “College #1” declined, while the geographic distribution of cases broadened. Subsequent cases emerged from a secondary school (*n* = 1), a healthcare facility (*n* = 3), “College #2” (*n* = 6), a multinational company (*n* = 1), and a household (*n* = 3). These latter cases were dispersed across western Switzerland, and it was not possible to confirm whether these locations fell within the catchment.

Following these observations, we found that while WBS excels at identifying large-scale, early outbreak dynamics, it may be less sensitive for detecting smaller, contained transmission events, which potentially span multiple catchment areas. This underscores the need for future monitoring efforts to incorporate surrounding catchments potentially impacted by outbreaks, improving response efforts, and capturing cases that stray from the initial points of transmission.

## Outlook and Limitations

Our study underscores the potential of WBS to provide insights into measles outbreaks, complementing traditional measles surveillance or offering an alternative surveillance approach when clinical surveillance is limited or absent. Notably, the minimum threshold of cases required for reliable detection remains uncertain, as evidenced by the undetectable viral loads during the reduced case incidence rate. Furthermore, spatial factors, including residence and mobility within and between wastewater treatment catchment areas, may complicate wastewater detection. More detailed demographic and mobility data may help to better elucidate the relationships between clinical and wastewater-based surveillance. Finally, while the use of dPCR allowed for sensitive and specific detection of the measles WT virus, we did not detect the VA virus in this study despite reported vaccination campaigns. The inability of dPCR to quantify low viral loads underlines the potential of technological or methodological advancements to improve sensitivity. Future implementation of WBS for measles could enhance surveillance strategies but would benefit from improved integration with geospatial and epidemiological data including vaccination and shedding rates. Such an approach could further improve outbreak detection and management, including the potential to develop predictive frameworks.

## Supplementary Material



## Data Availability

All data produced in the present study are available at the following DOI: https://doi.org/10.25678/000E26. Data on wastewater viral loads are included in the Supporting Information. Clinical case, timing, and transmission chain analysis are publicly available in Cassini, Cobuccio, et al.^7^ at https://doi.org/10.2807/1560-7917.ES.2024.29.22.2400275 (accessed April 30, 2025). Figures representing clinical case data are made public here: https://www.cobuccio.me/transmission_chains_colorblind_proof (accessed: 14.03.2025) and https://www.cobuccio.me/measles_epicurve.html (accessed: 14.03.2025). Associated preprint: Gan, C.; Pitton, M.; de Korne-Elenbaas, J.; Cobuccio, L.; Cassini, A.; Ort, C.; Julian, T. R. Retrospective Wastewater Based Tracking of Measles Outbreak in Canton of Vaud, Switzerland: January–March 2024; medRxiv10.1101/2025.03.11.25322836 (accessed April 8, 2025).

## Notes

are in the Supporting Information. The Supporting Information is available free of charge at https://pubs.acs.org/doi/10.1021/acs.estlett.5c00244.

Additional information on the validation of the duplex assay including details on primer and probe sequences, primer and probe concentration optimization, performance comparisons from single-plex to duplex, limit of detection and limit of quantification analysis, raw wastewater viral load values, positive controls used, and analysis parameters for classifying dPCR partitions (PDF)
