# Supplementary material for: Retrospective Wastewater Tracking of Measles Outbreak in Western Switzerland in Winter 2024
Source: Environ Sci Technol Lett. 2025 May 22;12(6):689–94. doi: 10.1021/acs.estlett.5c00244 (PMC12160055; doi:10.1021/acs.estlett.5c00244)
Supplement: Supplementary file 1 [file ez5c00244_si_001.pdf]

Supplemental Material

Retrospective Wastewater Tracking of Measles Outbreak in Western Switzerland in Winter 2024

Charles Gan[1†], Melissa Pitton[1†], Jolinda de Korne-Elenbaas[1], Ludovico Cobuccio[2,3], Alessandro Cassini[4], Christoph Ort[1], Timothy R. Julian[1,5,6*]

[1] Eawag, Swiss Federal Institute of Aquatic Science and Technology, Dübendorf 8600, Switzerland

[2] Cantonal Doctor Office, Public Health Department, Lausanne 1014, Switzerland

[3] Infectious Diseases Service, Lausanne University Hospital, Lausanne 1005, Switzerland

[4] Cantonal Office of Health, Geneva 1207, Switzerland

[5] Swiss Tropical and Public Health Institute, Allschwil 4123, Switzerland

[6] University of Basel, Basel 4001, Switzerland

[†] Authors contributed equally

[*] **Corresponding author:**

Timothy R. Julian, email: tim.julian@eawag.ch

4 Text, 3 Tables, 5 Figures

Text

Text S1. Comparison of primer and probe concentrations

Primer and probe sequences were sourced from Wu et al.[1], where optimal performance was demonstrated using 900 nM primers and 250 nM probes. To evaluate whether reduced concentrations could yield comparable results, three serial dilutions of a positive control, comprising synthetic DNA from the measles B3 strain and extracted measles vaccine, were analyzed in parallel using two primer/probe concentration sets: 900/250 nM and 500/200 nM, respectively. Reduced primer and probe concentrations were evaluated in accordance with manufacturer recommendations, which advocate for minimizing oligonucleotide concentrations to reduce potential interaction complexity within the reaction. All measurements were performed in triplicate. Comparison between the two conditions showed no significant difference, with a correlation coefficient of $R^2 > 0.99$ and a slope of 0.96 for measles wild-type (WT) and an R2 > 0.99 and a slope of 0.99 for measles vaccine (VA) (Figure S1).

Text S2. Single-plex assay performance

Prior to multiplexing, we tested each target in single-plex assays to ensure the separation between positive and negative clusters, and we tested the linearity using synthetic positive controls. Each single-plex assay showed clear distinction between positive and negative clusters, and data was fitted to linear regression, leading to R^2 values equal or greater than 0.99 for each target (Figure S).

Text S3. Comparison of single-plex to duplex assays performance

To ensure that primer and probe interactions were minimal, quantification of positive material was compared between single-plex and duplex for both duplex assays (Figure S). To accomplish this, a mix of measles B3 strain synthetic DNA and measles vaccine was 10-fold diluted three times and then measured in single-plex and duplex for both duplexes in technical replicates. The concentrations of individual replicates were averaged, and paired t-tests were performed to determine whether significant difference existed between single-plex and duplex concentrations for each dilution. Significance was set to $p = 0.05$. We observed that concentrations did not significantly differ between single-plex and duplex for measles WT/VA (Figure S) ($p > 0.05$ for each pairwise comparison).

Text S4. Limit of Detection (LoD) and Limit of Quantification (LoQ) of duplex assays

The LoD was determined empirically using multiple replicates of dilutions at low concentrations and fitting a dose-response model adapted for qPCR as described by Klymus et al. (2020)[2], where fit conditions were set to "best" or the model with the lowest residual error. The LoD is influenced by the number of replicates analyzed per sample, as the chances of detection improve with a higher number of replicates. Consequently, LoD values determined for a single replicate are extended to estimate those for varying numbers of replicates (e.g., one, two, three, four, five, and eight replicates) as shown in Figure S4. The detection probability associated with each replicate count was expressed as a single-replicate detection probability by calculating the nth-root of the non-detection probability (e.g., for 95% detection: $1 - (0.05)^{(1/n)}$, where n represents the number of replicates). Conforming to the methods used in this study, we implemented a 2-replicate LoD since all wastewater extracts were run in duplicate.

To determine the LoQ, multiple replicate dilutions at moderate-to-low concentrations were measured, with the resultant data fit to a power model, CoV = a × C_{dpcr}^{-k}, where CoV is the coefficient of variation, a is the scaling factor, C_{dpcr} is the concentration measured by dPCR, and k is the power relationship exponent (Figure S5). Both LoD and LoQ models were fitted with experimental data generated by measuring a minimum of 20 replicates at decreasing concentrations ranging from 110 to 6.4 gc/reaction for measles wild type (B3 gBlock®) and 40 to 8.4 gc/reaction for measles vaccine.

Both LoD and LoQ values were first expressed in gc/reaction and then multiplied by 1’200 (reactions/liter), which is a conversion factor to units of gc/L wastewater (gc/L_{ww}). The conversion factor is specific to our nucleic acid extraction, dilution factor, and digital PCR detection protocols (Equation 1).

$$\left[\frac{gc}{L_{ww}}\right] = \left[\frac{gc}{reaction}\right] * 3\ (dilution) * \frac{1}{5\frac{\mu L\ extract}{reaction}} * 80\ \mu L\ extracted * \frac{1}{40 mL\ wastewater} * \frac{1000 mL}{1 L} \quad (1)$$

Tables

Table S1. Measles wild type RNA concentration, viral load, and inhibition in Lausanne wastewater. C: concentration. AVG: mean. MSLS: measles. STD: standard deviation. WT: wild type. CV: coefficient of variation. NA: not applicable due to zero value. “-“: not measured.

Date	Flow Rate (m^3)	C_{AVG} MSLS WT [gc/L_{ww}]	C_{STD} MSLS WT [gc/L_{ww}]	AVG of MSLS WT load (gc/d/person)	STD of MSLS WT load (gc/d/person)	CV of MSLS WT load (%)	Inhibition (%)
2024-01-02	233900	0	0	0	0	NA	-
2024-01-04	118687	0	0	0	0	NA	-
2024-01-05	160970	0	0	0	0	NA	-
2024-01-06	117133	0	0	0	0	NA	-
2024-01-07	112260	0	0	0	0	NA	-
2024-01-08	101322	0	0	0	0	NA	-
2024-01-10	108052	0	0	0	0	NA	-
2024-01-12	93961	0	0	0	0	NA	-
2024-01-13	91343	0	0	0	0	NA	-
2024-01-14	93173	0	0	0	0	NA	-
2024-01-16	106184	0	0	0	0	NA	-
2024-01-18	371137	0	0	0	0	NA	-
2024-01-19	167938	0	0	0	0	NA	-
2024-01-20	129327	0	0	0	0	NA	-
2024-01-21	117022	0	0	0	0	NA	-
2024-01-22	208803	0	0	0	0	NA	-
2024-01-24	121704	0	0	0	0	NA	3
2024-01-26	105482	0	0	0	0	NA	-
2024-01-27	98898	0	0	0	0	NA	-4
2024-01-28	97311	0	0	0	0	NA	-
2024-01-30	93713	8.40E+03	8.49E+03	3.28E+06	3.31E+06	101%	-
2024-02-01	92045	1.92E+04	9.76E+03	7.36E+06	3.74E+06	51%	3
2024-02-02	89750	8.25E+03	1.48E+03	3.09E+06	5.55E+05	18%	-
2024-02-03	86453	7.50E+03	4.24E+02	2.70E+06	1.53E+05	6%	-4
2024-02-04	85837	1.77E+04	7.21E+03	6.33E+06	2.58E+06	41%	-
2024-02-05	87525	2.18E+04	5.30E+03	7.93E+06	1.93E+06	24%	2
2024-02-07	110160	1.02E+04	4.24E+03	4.68E+06	1.95E+06	42%	-
2024-02-09	92602	3.30E+04	1.91E+04	1.27E+07	7.37E+06	58%	8
2024-02-10	96657	1.02E+04	2.55E+03	4.11E+06	1.03E+06	25%	-

2024-02-11	96108	0	0	0	0	NA	-4
2024-02-13	85468	0	0	0	0	NA	-
2024-02-15	82418	0	0	0	0	NA	-2
2024-02-16	81992	0	0	0	0	NA	-
2024-02-18	78897	0	0	0	0	NA	-1
2024-02-19	95700	0	0	0	0	NA	-
2024-02-21	82531	0	0	0	0	NA	9
2024-02-23	231231	0	0	0	0	NA	-
2024-02-24	111999	0	0	0	0	NA	-
2024-02-25	92632	0	0	0	0	NA	-
2024-02-27	106912	0	0	0	0	NA	-
2024-02-29	95245	0	0	0	0	NA	-
2024-03-01	92043	0	0	0	0	NA	-
2024-03-02	89262	0	0	0	0	NA	-
2024-03-03	85238	0	0	0	0	NA	-
2024-03-04	84652	0	0	0	0	NA	-
2024-03-06	85544	0	0	0	0	NA	-
2024-03-08	84507	0	0	0	0	NA	-
2024-03-09	81314	0	0	0	0	NA	-
2024-03-10	86489	0	0	0	0	NA	-
2024-03-12	85144	0	0	0	0	NA	-
2024-03-14	89249	0	0	0	0	NA	-
2024-03-15	123496	0	0	0	0	NA	-
2024-03-16	87515	0	0	0	0	NA	-
2024-03-17	119503	0	0	0	0	NA	-
2024-03-18	146879	0	0	0	0	NA	-
2024-03-20	92613	0	0	0	0	NA	-
2024-03-22	93594	0	0	0	0	NA	-
2024-03-23	91342	0	0	0	0	NA	-
2024-03-24	87584	0	0	0	0	NA	-
2024-03-26	90254	0	0	0	0	NA	-
2024-03-28	112914	0	0	0	0	NA	-
2024-03-29	87805	0	0	0	0	NA	-
2024-03-30	81727	0	0	0	0	NA	-
2024-03-31	126624	0	0	0	0	NA	-

65

Table S2. Measles Wild Type (WT) and Vaccine (VA) primers and probes sourced from Wu et al[1] with modified dyes and quenchers. WT: wild type. VA: vaccine. /5Cy55/: Freedom Cy®5.5 dye modification from IDT (Iowa, USA). /5Cy5/: Freedom Cy®5 dye from IDT (Iowa, USA). /3IAbRQSp/ - Iowa Black® RQ Dark Quencher from IDT (Iowa, USA).

Oligo Type	Sequence (5' to 3')
Forward Primer	AATGAAAAACTGGTGTTCTACAA
Reverse Primer	GGTGATGCTCATATAAACAACAC
Probe – WT	/5Cy55/TATCCAGCGGTATCAGATTAACTGCATTGCA**/3IAbRQSp/**
Probe – VA	/5Cy5/TATCGAGCG**/TAO/**GTATCAGATTAACCGCATTGCA**/3IAbRQSp/**

Table S1. Characteristics of measles WT and VA positive controls used.

Positive Material Type	Sequence/Genbank #	Details
Measles B3 strain gBlock®	GTCCCTGCCCCTAGGTGTTGGCAGATCCACAGC<br>AAAACCCGAAGAACTCCTCAAGGAGGCCACTGA<br>GCTTGACATAGTTGTTAGACGTACAGCAGGGCT<br>CAATGAAAAACTGGTGTTCTACAACAACACTCCA<br>CTAACTCTCCTCACACCTTGGAGAAAGGTCCTG<br>ACAACAGGGAGTGTCTTCAACGCAAATCAAGTG<br>TGCAATGCGGTTAATCTGATACCGCTGGATACC<br>CCGCAGAGGTTCCGTGTTGTTTATATGAGCATC<br>ACCCGTCTTTCAGATAACGGGTATTACACTGTTC<br>CTAGAAGAATGCTGGAATTCAGATCAGTCAATG<br>CAGTGGCCTTCAACCTGCTGGTGACCCTTAGGA<br>TTG	Nucleotide 3656-4025
Measles Schwarz vaccine strain	Genbank # GCA_031113345.1	Whole Genome

Figures

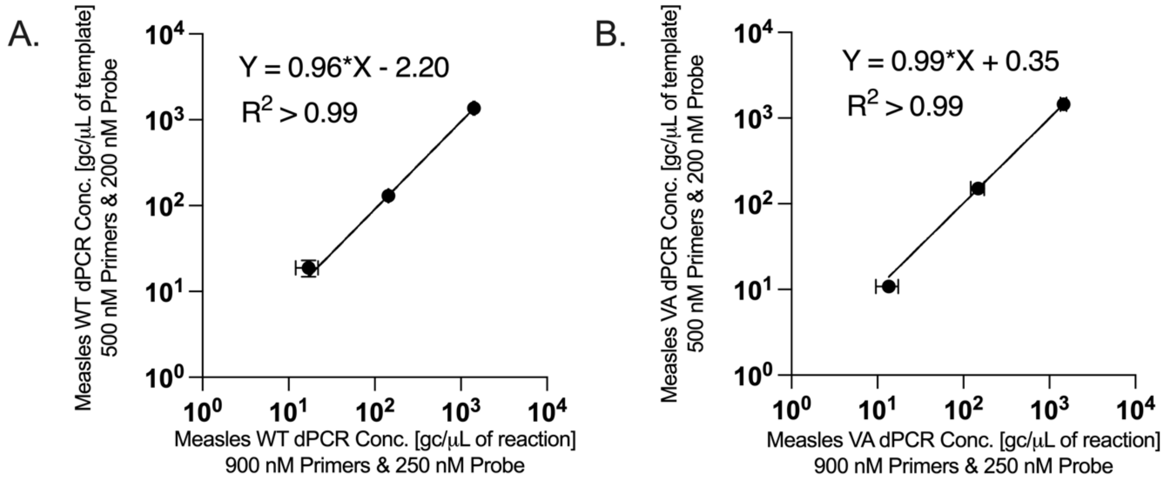

Figure S1. Quantification comparison between 900 nM primers with 250 nM probes and 500 nM primers with 200 nM probes using positive controls with measles WT and measles VA. Reduced primer and probe concentrations were tested to assess compatibility of an existing primer probe sequence set[1] with a different digital PCR system and modified dyes and quenchers. (A and B) Vertical axis represents the digital PCR (dPCR) concentration measured using the primer concentration of 500 nM with probe concentration of 200 nM. Horizontal axis represents the dPCR concentration measured using the primer concentration of 900 nM and probe concentration of 250 nM. Individual points represent the empirical average of triplicates with error bars representing the standard deviation. (A) For measles WT, linear regression analysis yielded an $R^2 > 0.99$ and a slope of 0.96. (B) For measles VA, a linear regression analysis yielded an $R^2 > 0.99$ and a slope of 0.99.

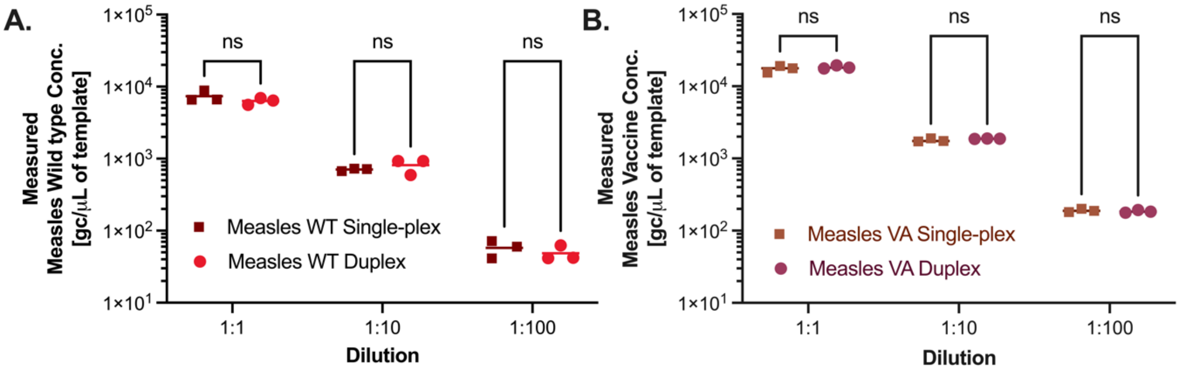

Figure S2. Quantification comparison between single-plex assays and duplexes using positive controls. Horizontal axes show three 10-fold dilutions of the positive material. Y-axes indicate the measured concentrations using dPCR and expressed in genome copies (gc) per microliter (µl) of template. Within each dilution category, individual squares and circles represent replicate measurements of the same sample. Viral targets are specified with distinct colors and sorted by panel: measles WT in panel A and measles vaccine in panel B. Statistical significance was inferred using multiple paired t-tests. ns: not significant. All p-values were greater than 0.24 with significance threshold established as $p < 0.05$.

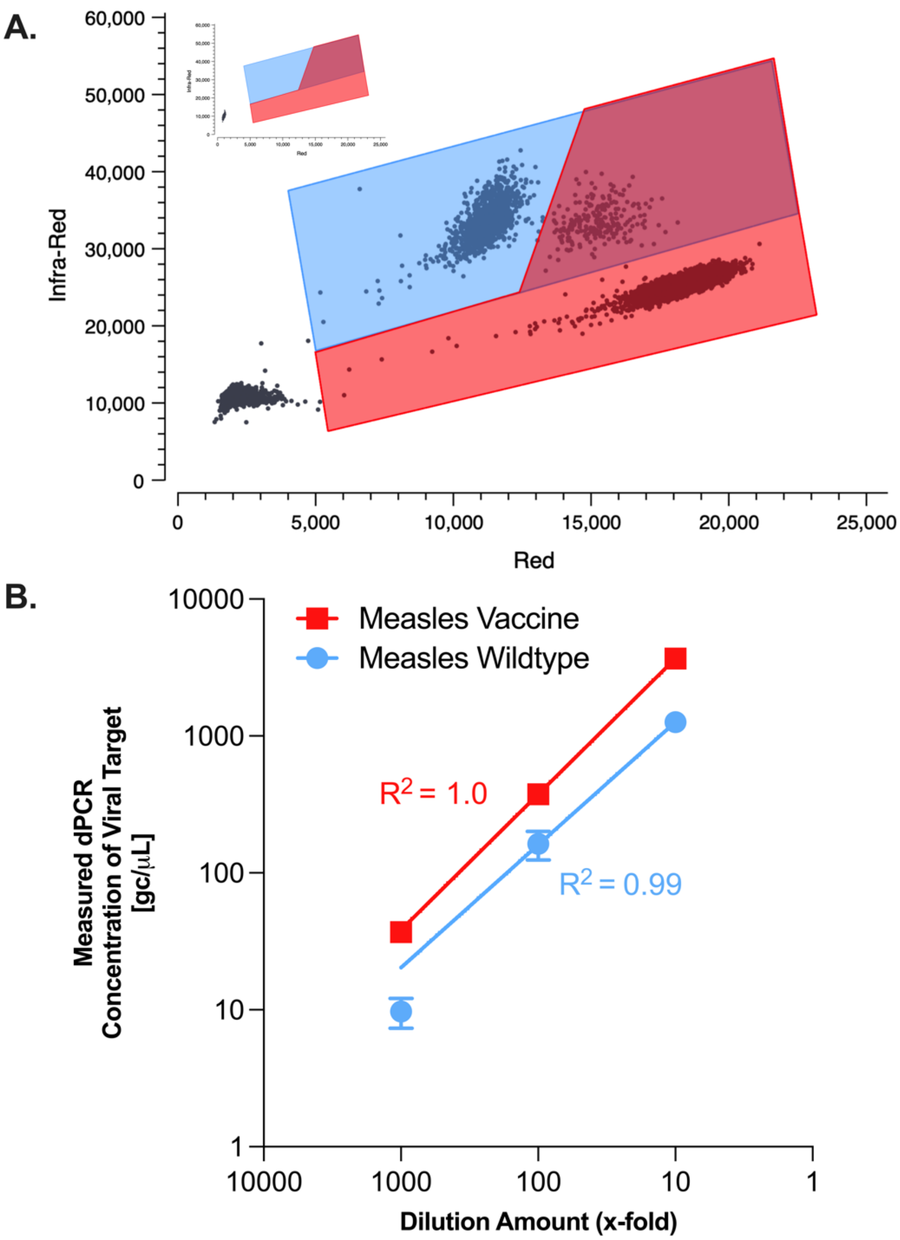

Figure S3. Validation of single-plex dPCR assays. (A) Two dimensional representations of partition classification. Horizontal axis shows fluorescent intensity in the red channel. Vertical axis displays the fluorescent intensity in the infrared channel. Each dot represents a single partition. Positive partitions are classified by polygon gating where the blue polygon contains partitions with measles WT and the red polygon contains partitions with measles VA. (B) Linearity testing for duplex assay. Red line represents the concentration of measles VA and blue line represents the concentration of measles WT. Horizontal axis indicates the fold dilution factor (10-fold dilutions). Vertical axis illustrates the measured concentration using dPCR and it is expressed in genome copies (gc) per microliter (µl) of template. Each dilution was measured in technical triplicates. Data was fitted to linear regression and R^2 values are displayed for each target ($R^2 = 1.0$ for measles VA and $R^2 = 0.99$ for measles WT).

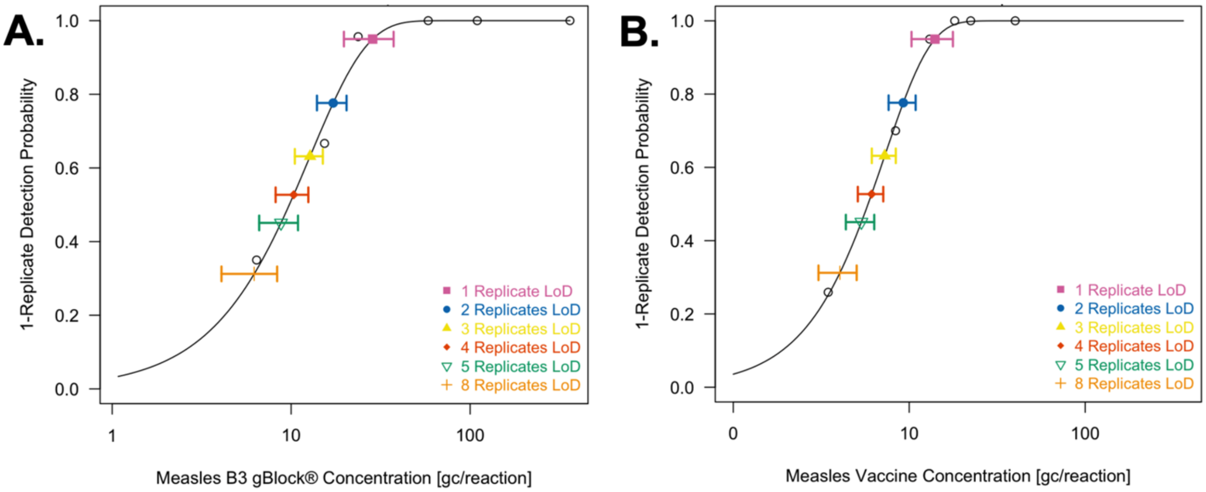

Figure S4. Modelled LoD of all targets measured using measles WT and VA duplex assay. Weibull Type-2, two-parameter detection probability curves show the likelihood of detecting each viral target at increasing concentrations, expressed as genome copies (gc) per reaction. Panels A and B represent different viral targets: (A) measles B3 gBlock® concentration and (B) measles VA concentration. Each colored line and symbol represent the limit of detection (LoD) for different numbers of replicates (1 to 8). The probability of detection approaches 1.0 as the target concentration increases, with LoD improving (shifted left) as replicate numbers increase. Error bars indicate the asymptotical Wald-type confidence intervals for each LoD threshold.

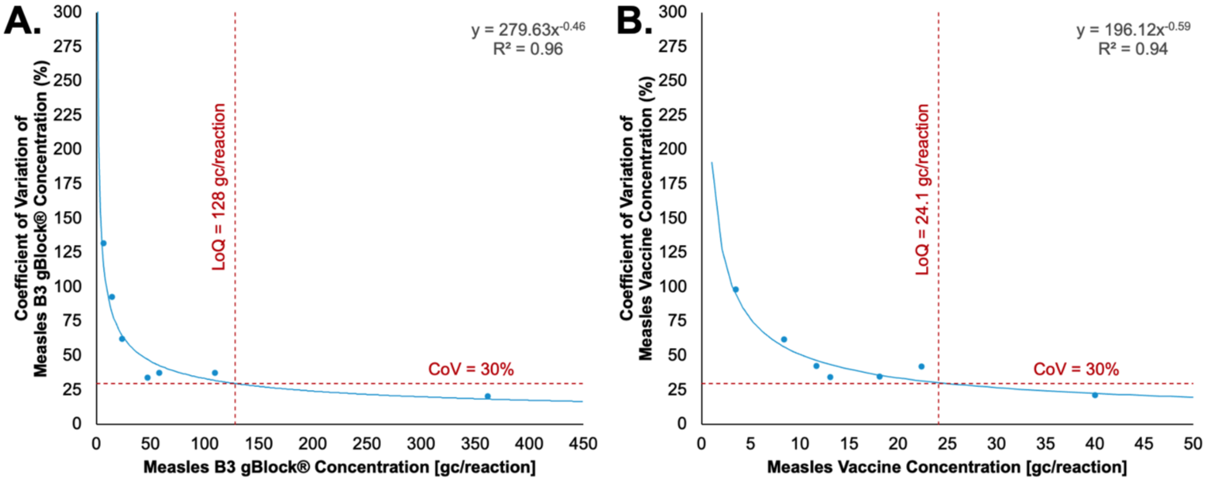

Figure S5. Modelled LoQ of all targets measured using measles WT and VA duplex assay. (A and B) Viral target concentration is represented on the horizontal axis in gene copies per reaction. Coefficient of variation at each concentration is represented on the vertical axis. A power law model is fitted through experimental data. The panels A and B represent the different viral targets. (A) Measles B3 gBlock® concentration: LoQ at 128 gc/reaction, with a model fit equation $y = 279.63x^{-0.46}$ and $R^2 = 0.96$ (B) Measles vaccine concentration: LoQ at 24.1 gc/reaction, with a model fit equation $y = 196.12x^{-0.59}$ and R^2=0.94 CoV decreases as target concentration increases. The dashed horizontal red line indicates the 30% CoV threshold for reliable quantification, and the vertical dashed red line represents the interpolated LoQ for each target.

Supplementary References

(1) Wu, J.; Wang, M. X.; Kalvapalle, P.; Nute, M.; Treangen, T. J.; Ensor, K.; Hopkins, L.; Poretsky, R.; Stadler, L. B. Multiplexed Detection, Partitioning, and Persistence of Wild-Type and Vaccine Strains of Measles, Mumps, and Rubella Viruses in Wastewater. *Environ Sci Technol* **2024**, *58* (50), 21930–21941. https://doi.org/10.1021/acs.est.4c05344.

(2) Klymus, K. E.; Merkes, C. M.; Allison, M. J.; Goldberg, C. S.; Helbing, C. C.; Hunter, M. E.; Jackson, C. A.; Lance, R. F.; Mangan, A. M.; Monroe, E. M.; Piaggio, A. J.; Stokdyk, J. P.; Wilson, C. C.; Richter, C. A. Reporting the Limits of Detection and Quantification for Environmental DNA Assays. *Environmental DNA* **2020**, *2* (3), 271–282. https://doi.org/10.1002/EDN3.29.
